# P01-07 Promoting physical activity in Finnish early childhood education and care, and the implementation of national recommendations of physical activity for early childhood

**DOI:** 10.1093/eurpub/ckac095.007

**Published:** 2022-08-29

**Authors:** Arja Sääkslahti, Nina Korhonen, Tuija Tammelin

**Affiliations:** 1 Faculty of Sport and Health Sciences, University of Jyväskylä, Jyväskylä, Finland; 2 Joy in Motion programme, National Agency of Education, Helsinki, Finland; 3 LIKES Research Centre for Physical Activity and Health, Jyväskylä, Finland

**Keywords:** preschool-aged, children, programme

## Abstract

Joy in Motion is a nationwide physical activity and well-being programme aimed at early childhood education and care (ECEC) launched in 2015 in Finland. The aim is to enable every child to be physically active and enjoy physical activity every day. Latest updates of the Finnish recommendations for physical activity in early childhood was published in 2016. The key message in the recommendations is Joy, play and doing together. Daily physical activity is just as important for children's well-being as healthy nutrition and sufficient sleep and rest. Presentations discuss the current state of the programme with more than 2200 registered early education units and provides concrete examples of the promotion work in practice.

